# The Food We Eat

**Published:** 1876-05

**Authors:** 


### EDITORIAL. ETC.

---

*The Food we Eat.*—A valuable article appeared in the *Boston Journal of Chemistry* upon "Wheat and the Deficiencies ot White Flour as a Nerve Food," which has been supplemented by an important lecture by the author, Dr. Cutler, of Massachusetts, upon the general subject, with special view to answering the question, "Is flour (white) our proper food?" Dr. Cutler has for a long time been engaged in extended researches upon the "effects of flour-eating upon the human race." Among the points made in his lecture is that flour is the only impoverished food used by mankind—impoverished by the withdrawal of the tegumentary portion of the wheat, leaving the internal starchy or white portion. Flour is mostly starch, 68.7 per cent. The human body contains at least twelve elements besides those of starch. How then, it is asked, can flour be nutritious with about three elements, when it should contain fifteen elements in order to properly nourish and sustain the human body? Flour has less ginten than wheat. Ginten is the albuminoid principle corresponding to the albumen, fibrin and gelatine in the human body.

It is related in Kirke & Pagel's "Physiology" that dogs fed by Magendie on flour bread died in forty days; other dogs, fed on bread from whole wheat meal or flour flourished and throve. The three-fourths impoverishment of the mineral ingredients proved fatal to the first. The question is asked why should not mankind suffer in the same manner from living on impoverished food? The fact that wheat as an article of food, combined with fresh out door air life, is capable of producing and sustaining the highest type of physical manhood the world ever saw is illustrated by the history of the Roman empire in the time of Julius Cæsar, which was built up and maintained by soldiers whose main article of food was wheat. Dr. Cutler also holds that it is probable that the present prevalence of decaying teeth, and that premature grayness or baldness are partly due to the exclusive and universal use of white flour. Hair contains ten per cent. of sulphuroid, but there is no sulphur or sulphuric acid in flour. A food, to be food, must contain in proper quantities all ingredients found in the tissues, hair, teeth, &c. In addition to this, white flour for half a century has been regarded as one cause of constipation. It has been proved that whole wheat meal regulates the bowels by giving the system nerve food to "run," so to speak the digestive functions. While wheat contains, in normal proportions, every element which belongs to the tissues and organs, white flour made from wheat is a substance weakened, deteriorated and impoverished, and history shows that people eating it are more subject to tissue-wasting disease, (consumption, &c.) than ever before. "Why, then," it is asked' "not use the whole of the original wheat, ground or reduced to a uniform condition, without loss or injury to the food elements, with its native normal balance, and quality of mineral ingredients in a soluble and assimilable form, as Liebig and others advocate; and such as it is demonstrated undeniably and in controvertibly, by facts of history, to be capable of producing the highest type of physical manhood the world ever saw. Why raise a pale, feeble, nervous and small-sized race of people upon flour because flour bread looks white and light, and therefore is considered nice?" But this attractive whiteness comes from starch, and the whiter the bread the more starch it contains, and, of course, the less nutrition. The whiteness of flour is regarded by Dr. Cutler as an outward sign of the starvation and death within. "What is wanted," says an intelligent writer on this subject, "is a wholesome, healthful, nourishing wheat food—a whole wheat flour in the fullest and broadest sense of the term. It should be made from the choicest and ripest wheat, wholly bran or cortical portion and all reduced to a uniform fineness of quality and well put up for family use; and whoever will give his earnest and honest efforts to furnishing such a flour, and will keep its manufacture up to this high standard all the time, will confer a lasting benefit upon his race and generation, and find a remunerative market for all he can produce."

